# Organic-Solvent-Resistant Polyimide/Hydroxyapatite Mixed Matrix Membranes for Lysozyme Adsorption

**DOI:** 10.3390/ma16227210

**Published:** 2023-11-17

**Authors:** Junfen Sun, Hao Pang, Long Chen

**Affiliations:** State Key Laboratory for Modification of Chemical Fibers and Polymer Materials, College of Material Science and Engineering, Donghua University, North People Road 2999, Shanghai 201620, China; 13262792919@163.com

**Keywords:** polyimide, hydroxyapatite, mixed matrix membranes (MMMs), crosslinking, lysozyme (LZ) adsorption

## Abstract

This work reports new mixed matrix membranes (MMMs) for the adsorption of enzymes from organic solvents. In this work, polyimide/hydroxyapatite (PI/HAP) MMMs were prepared via phase inversion method and further crosslinked with 3-aminopropyl triethoxysilane (APTES). The chemical and structural stability of the crosslinked PI/HAP MMMs were improved and applied for lysozyme (LZ) adsorption in organic solvent. PI/HAP MMMs were crosslinked by changing the 3-aminopropyltriethoxysilane (APTES) concentration and crosslinking time. The optimal APTES crosslinking condition for PI/HAP MMMs is 6% of concentration for 8 h. The LZ adsorption performance was studied by changing solvent types. PI/HAP MMMs possessed a high LZ adsorption in organic-solvent-aqueous solutions, and the LZ adsorption capacity reached 34.1 mg/g. The MMMs had a high desorption capacity and recovery ability. The MMMs maintained 60% of their adsorption capacity and 58% of their desorption at the fourth cycle of adsorption and desorption. The MMMs provided a new technology for the purification and separation of enzymes or proteins by MMMs in organic solvents.

## 1. Introduction

Enzymes are well recognized as practical catalysts because of their tremendous potential application and are being increasingly exploited for asymmetric syntheses, transformations and industrial processes [1,2]. However, most enzymes could not be dissolved in water, and water sometimes leads to unwanted side reactions and degradation of common organic reagents. The thermodynamic equilibria of many processes are unfavorable in water, and sometimes it is difficult to recover products from water [3]. The proteolysis of enzymes could be overcome and the stability of enzymes improved by using organic solvents as reaction media. The conformational flexibility of enzymes keeps very well in aqueous-organic mixtures and even in anhydrous organic solvents that could not give rise to the denaturation of enzymes. Recently, the use of enzymes [4] or modified enzymes [5] to improve biocatalysis in organic solvents has been developing very fast.

It was previously widely used to employ adsorbers in liquid mixtures to remove proteins or enzymes based on their chemical and physical properties [6]. The adsorbers could be classified as particles, microspheres and membranes, among other things in the field of solid extraction. Recently, mixed matrix membranes (MMMs) have been studied by many researchers because MMMs combine the advantages of membrane technology and particles. Additionally, MMMs provide a low pressure drop, high throughputs, a high efficiency, an easy scale-up, etc. The adsorption-based separation behavior involves the preferential participation of adsorbate molecules moving from the liquid phase onto the substrate surface [7]. Adsorptive MMMs incorporate adsorptive particles into the polymeric porous matrix where the interactions between enzymes or proteins and adsorptive particles happen. So far, different adsorptive particles, such as ion-exchange resins [8,9], amino-functionalized silicon dioxide (NH_2_−SiO_2_) [10], activated carbon (AC) [11] and hydroxyapatite (Ca_5_ (PO_4_)_3_(OH), HAP) [12], were embedded into polymeric matrixes to adsorb enzymes or proteins. Modified HAP [13,14,15] and other phosphates [16,17,18] are also important and were used in biomedical applications. Previously, we prepared MMMs by incorporating HAP into different polymer matrixes (polyether sulfone (PES) [19], chitosan (CS) [20], polyvinylidene fluoride (PVDF) [21]), and those MMMs showed good bovine serum albumin (BSA) and lysozyme (LZ) adsorption and desorption properties in an aqueous system. MMMs combined a good adsorption property of HAP with a high productivity of the filtration membrane. Other polymers, such as ethylene vinyl alcohol copolymer (EVAL) [22], poly(ether imide) (PEI) [23], polyimide (PI) [24], etc., were also applied to prepare MMMs. PI is an excellent membrane material due to its high chemical and thermal stability [25]. In recent years, PI membranes combining different inorganic fillers (zeolite, metal-organic framework (MOF), zeolitic imidazolate framework (ZIF), carbon nanotube, etc.) were applied in gas separation, such as CO_2_/CH_4_ [26], propylene/propane (C_3_H_6_/C_3_H_8_) [27], H_2_/CO_2_, H_2_/N_2_, etc. [28]. Recently, they were also applied in dye/salt separation [29] as well as in lysozyme (LZ) and cholesterol separation [30] in organic solvent. The organic-solvent-resistant performance and stabilization of PI membranes [31] and PI-based MMMs [30] in organic solvents can be further improved via the chemical crosslinking of the PI matrix. Different crosslinking agents, such as aliphatic diamines (ethylene diamine (EDA) [32], 1,6-hexanediamine (HDA) [24], hexamethylenediamine (HMDA) [33], etc.) and silicon coupling agent ((3-isocyanatopropyl) triethoxysilane [34]), were used to crosslink PI molecular chains.

In this work, solvent-resistant MMMs for the adsorption of enzymes or proteins in organic solvents was developed and demonstrated. PI (P84) was used as a polymer matrix, which was crosslinked with 3-aminopropyltriethoxysilane (APTES), and therefore the MMMs became resistant to organic solvents. HAP was used as adsorption particles and was embedded in the PI matrix to prepare crosslinked PI/HAP MMMs. Subsequently, the crosslinked PI/HAP MMMs were used for the adsorption of LZ in N,N-dimethylformamide (DMF), dimethyl sulfoxide (DMSO) and N-methyl-2-pyrrolidinone (NMP). LZ plays an important role in mammalian bodies and is widely used in the medical and biological fields. LZ was used as a target enzyme in this work. The preparation process of crosslinked PI/HAP MMMs is simple, non-toxic and low-cost. A good adsorption performance of crosslinked PI/HAP MMMs provides a wide application as an adsorptive membrane in organic solvents.

## 2. Materials and Methods

### 2.1. Materials

Hydroxyapatite (HAP, d = 40 nm) was purchased from Nanjing Emperor Nano Material Company (Nanjing, China). Polyimide, type P84 (325 mesh, STD) was purchased from HP Polymer GmbH (Lenzing, Austria). Dimethylformamide (DMF), dimethyl sulfoxide (DMSO), N-methyl-2-pyrrolidone (NMP), 3-aminopropyltriethoxysilane (APTES) and Lysozyme (LZ) were bought from Sinopharm Chemical Reagent Company (Shanghai, China). All reagents used in the preparation and in the adsorption studies were of analytical grade. All used water was deionized.

### 2.2. Membrane Preparation

#### 2.2.1. Preparation of PI/HAP MMMs

PI/HAP MMMs were prepared by phase-inversion technique. DMF was used as the solvent to dissolve PI, and pure water was used as a coagulation bath. PI and HAP were dried in the vacuum oven (DZF-6020, Shanghai Sumsung Laboratory Instrument Co., Ltd., Shanghai, China) at 60 °C for 24 h. PI dope solution was prepared by dissolving 20 wt% PI in DMF for 10 h, and 20 wt% HAP particles were added in PI dope solution to obtain the mixed solution. The mixed solution was stirred for another 12 h and left for degassing for 12 h. After the removal of gas bubbles, the mixed solution was cast on glass substrates, and the membrane was immersed in a water bath where phase inversion occurred. After 2 h, the membrane was placed in a fresh water bath and left for 24 h to ensure sufficient removal of the solvent.

#### 2.2.2. Crosslinking of PI/HAP MMMs

**Figure 1:**
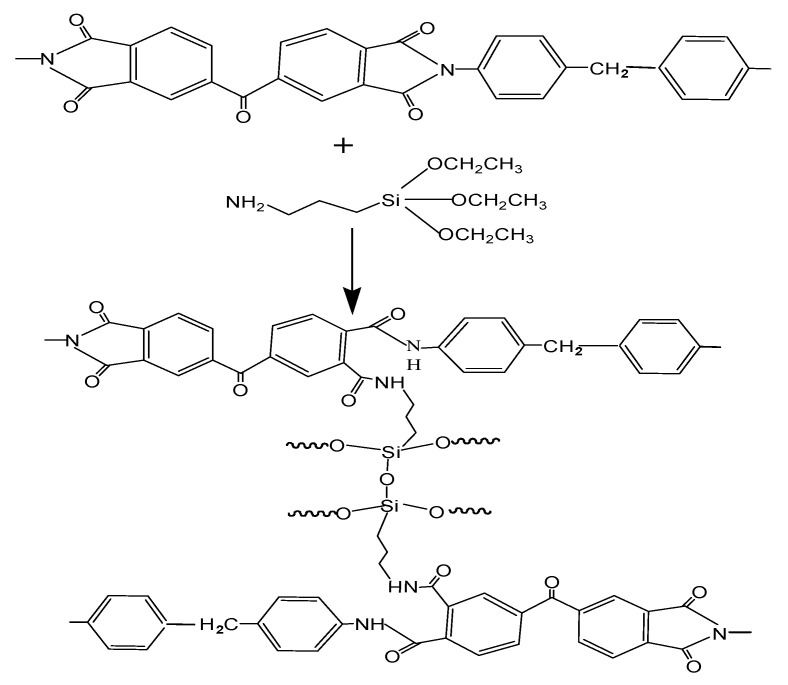
Schematic diagram of crosslinking of polyimide (PI) molecules with APTES.

PI/HAP MMMs were crosslinked by immersion into APTES/2-butanone (*w*/*w*) solution at 75 °C for 12 h and subsequently thoroughly washed with methanol (analytical grade, Sinopharm Chemical Reagent Company, Shanghai, China) to remove the residues of APTES. The APTES concentration (2, 4, 6, 8 and 10 wt%) and crosslinking time (2, 4, 6, 8, 10, 12 h) were changed to prepared crosslinked PI/HAP MMMs. The crosslinking reaction of PI by APTES is presented in Figure 1.

### 2.3. Membrane Characterization

The morphology of the membrane was observed by AFM (NanoScope IV, Veeco, Dallas, TX, USA) and SEM (Quanta-250 ESEM Co., Brno, Czech). The pure water flux of the membrane was tested by a dead-end filtration system, and the membrane was pre-compacted at 0.15 MPa for 30 min before the tests. The contact angle of the membrane was evaluated by a micro video contact angle measuring instrument (OCA40 Dataphysics Co., Stuttgart, Germany), and each membrane was captured six times to obtain an average value. The functional groups of the membrane were investigated by fourier transform infrared spectroscopy (FTIR, Nicolet Co., Vernon Hills, IL, USA). The tensile strength and elongation at break of the membrane were measured by a tensile testing machine (Chang-Zhou Textile Machine Co., Changzhou, China). The membranes were cut into rectangular splines (15 × 90 mm) and tested at a stretching rate of 20 mm/min.

### 2.4. Adsorption Experiments

LZ was used as an enzyme target in the adsorption study. Three different organic solvents (DMF, DMSO and NMP) were chosen, and the organic solvent/water ratios (90/10, 80/20, 70/30, 60/40 and 50/50, *v*/*v*) were used for the LZ adsorption of crosslinked MMMs.

The MMMs were dried at 30℃ under vacuum conditions for 10 h. MMMs measuring 15 × 15 mm were weighted and sealed in 5 mL 2 g/L LZ organic solvent–aqueous solution. The sealed solutions were shaken for 24 h at 25 °C. The concentration of LZ solution was determined with a UV−1800 spectrophotometer (SHIMADZU Company, Tokyo, Japan) at 281 nm, and the adsorption capacity of the MMMs was calculated as follows:

$$q=\frac{(C_{0}-C_{1})\times V}{W}$$

where *q* is the adsorption capacity (mg/g), *C*_0_ is the concentration of LZ original solution (mg/mL), and *C*_1_ is the concentration of LZ solution after adsorption (mg/mL). *W* is the weight of dry MMMs (mg), and *V* is the volume of LZ solution (mL).

The LZ desorption property was carried out by using NaCl (analytical grade, Sinopharm Chemical Reagent Company, Shanghai, China) aqueous solution (0.5 mol/L) at 25 °C for 24 h. The regeneration of PI/HAP MMMs was evaluated by four adsorption–desorption circle processes.

## 3. Results and Discussion

### 3.1. Effects of Crosslink Conditions on Pure Water Flux, Contact Angle, Mechanical Properties of MMMs

#### 3.1.1. Effect of APTES Concentration

**Figure 2:**
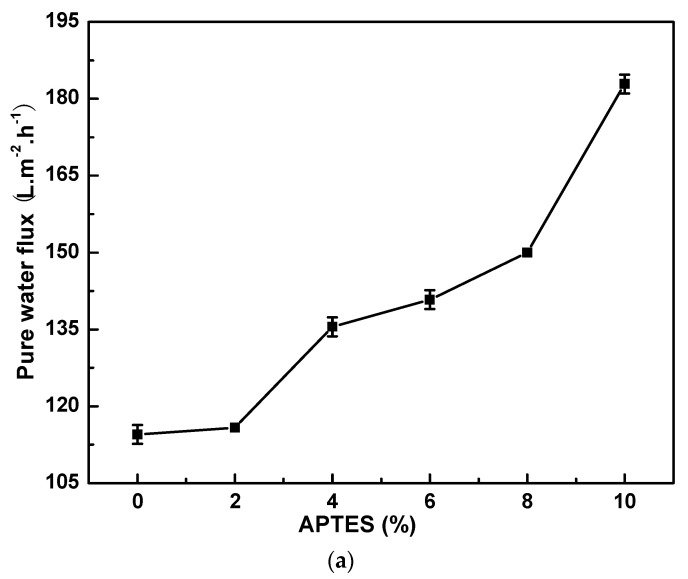

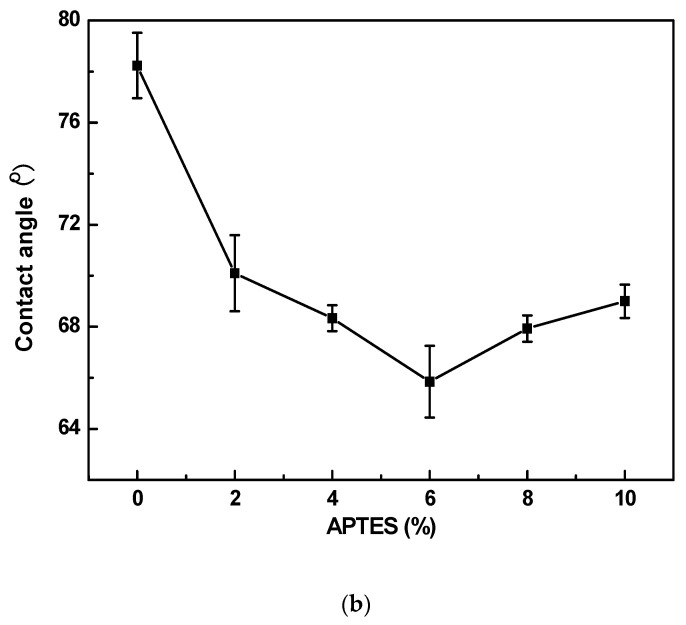
Effect of APTES concentration on (**a**) pure water flux and (**b**) contact angle of PI/HAP MMMs.

Figure 2 shows the effect of the APTES concentration on (a) pure water flux and (b) contact angle of PI/HAP MMMs. As shown in Figure 2a, the pure water flux increases with an APTES concentration increasing from 0 to 10%. As shown in Figure 2b, the contact angle decreases with an APTES concentration increasing from 0 to 6%, and reaches the minimum when the APTES concentration is 6%. MMMs become more hydrophilic after crosslinking with APTES. More APTES react with PI molecular chains when the APTES concentration increases. The imide group of PI becomes an amino group when PI reacts with APTES and the APTES molecule has an amino group, which improves the hydrophilicity of PI/HAP MMMs. The contact angle increases when further increasing the APTES concentration from 6 to 10%. There are more groups of Si−O−Si in PI molecular chains, which may restrain the hydrophilicity of PI/HAP MMMs.

**Figure 3:**
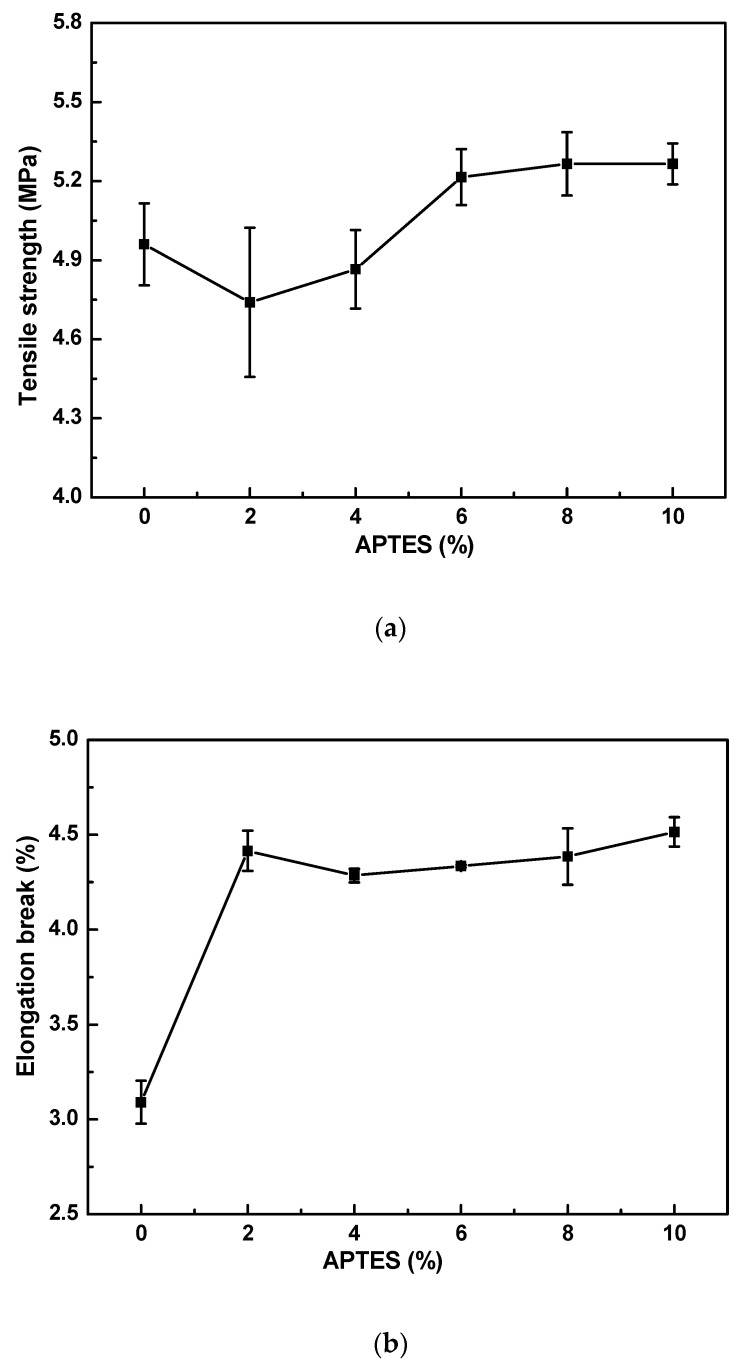
Effect of APTES concentration on (**a**) tensile strength and (**b**) elongation break of PI/HAP MMMs.

Figure 3 shows the effect of the APTES concentration on (a) tensile strength and (b) elongation break. As shown in Figure 3a, the tensile strength of MMMs is improved when the APTES concentration increases from 6 to 10%. Moreover, compared to non-crosslinked MMMs, the elongation break of crosslinked MMMs is also improved when the APTES concentration increases from 0 to 10%. PI molecular chains are not crosslinked completely by APTES when the APTES concentration is lower than 6%, and therefore the tensile strength and elongation break of MMMs are low at a low APTES concentration. PI molecular chains are crosslinked completely by APTES, and the full net structure of PI molecular chains forms when the APTES concentration is over 6%, which improves the mechanical property of MMMs. Thus, by comprehensive consideration, the optimal APTES concentration for MMMs is 6%.

#### 3.1.2. Effect of Crosslinking Time

**Figure 4:**
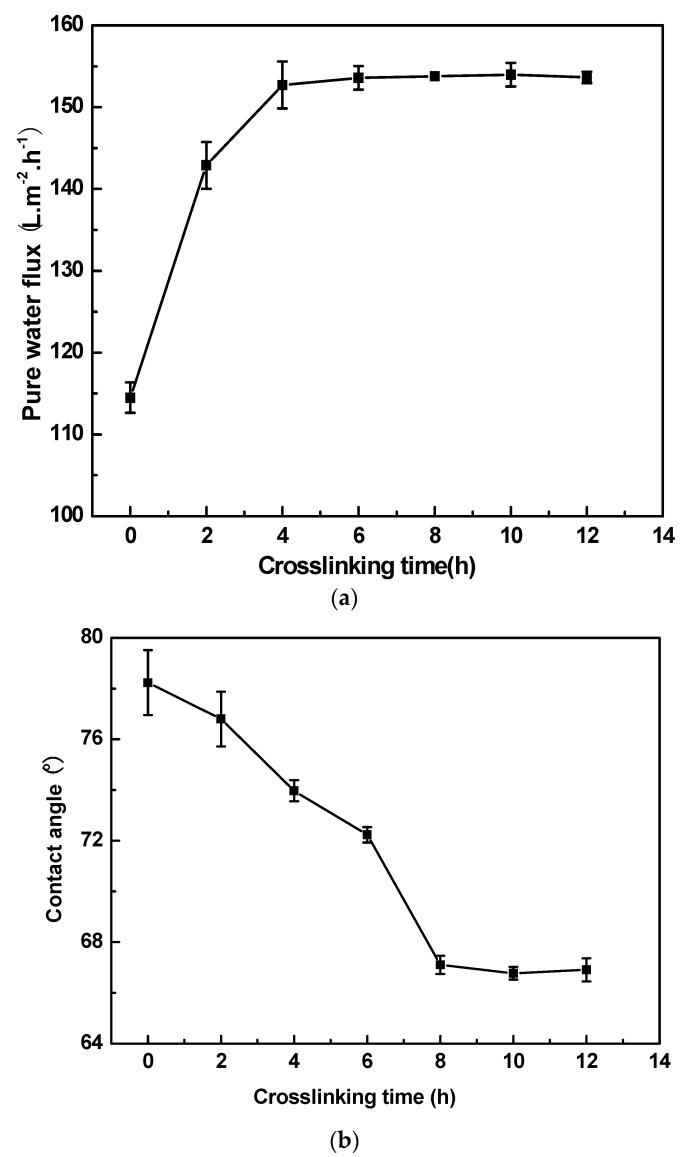
Effect of crosslinking time on (**a**) pure water flux and (**b**) contact angle of PI/HAP MMMs.

Figure 4 shows the effect of the crosslinking time on (a) pure water flux and (b) contact angle of PI/HAP MMMs. As shown in Figure 4a, the pure water flux increases with an increasing crosslinking time and reaches a stable value. This means that the stable MMMs’ structure forms after the MMMs are crosslinked for 4 h. As shown in Figure 4b, the contact angle decreases when increasing the crosslinking time, and is almost unchanging when the crosslinking time is more than 8 h. More imide groups of PI become amino groups when PI reacts with APTES with an increasing crosslinking time, and almost all of APTES is reacted when the crosslinking time is more than 8 h. No reaction proceeds when further increasing the crosslinking time. The hydrophilicity of PI/HAP MMMs is improved by extending the crosslinking time.

**Figure 5:**
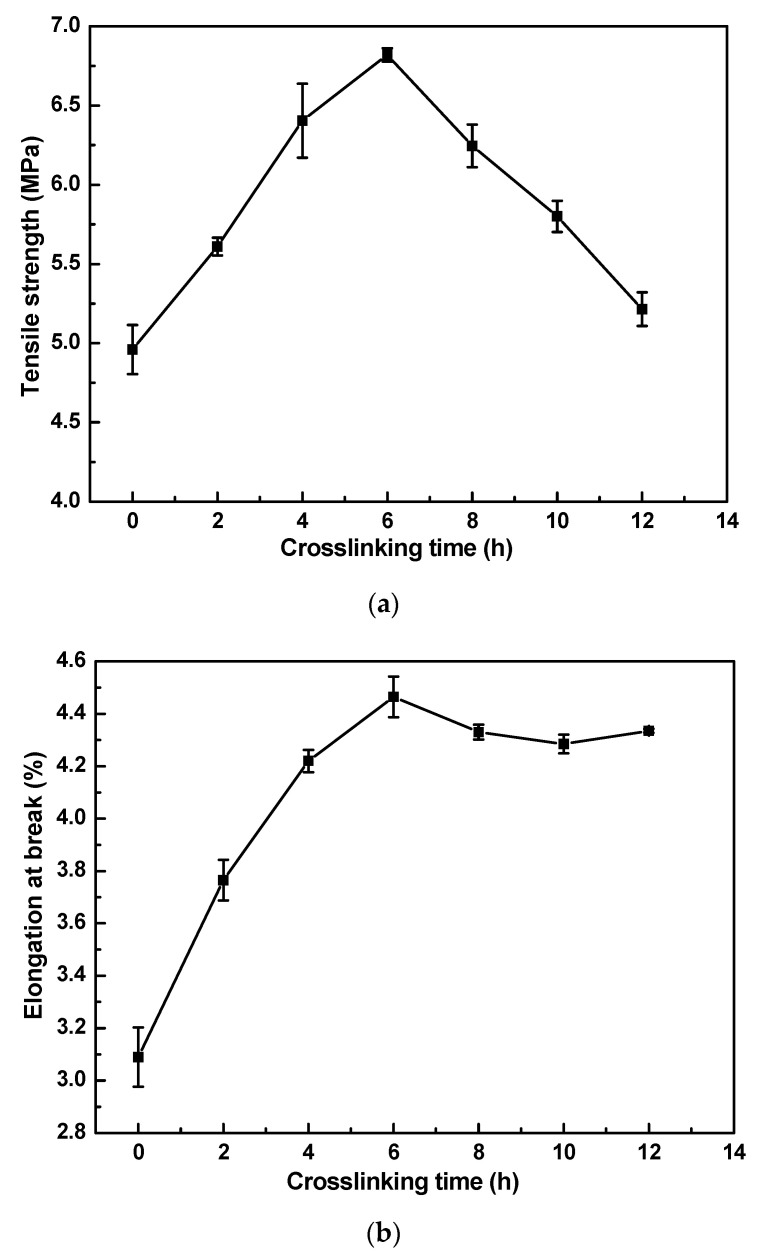
Effect of crosslinking time on (**a**) tensile strength and (**b**) elongation break of PI/HAP MMMs.

Figure 5 shows the effect of crosslinking time on (a) tensile strength and (b) elongation break. As shown in Figure 5a, the tensile strength of MMMs is improved when the crosslinking time increases from 0 to 6 h, and it reaches the maximum (MPa) at 6 h. Moreover, the elongation break of crosslinked MMMs is also improved when the crosslinking time increases from 0 to 6 h, compared to non-crosslinked MMMs. The crosslinking degree increases and the MMMs structure becomes more and more compact when extending the crosslinking time and when the crosslinking time is less than 6 h, which leads to an increased tensile strength and elongation break of MMMs. The branching reaction of PI molecular chains partly happens when further extending the crosslinking time when the crosslinking time is more than 6 h, which reduces the tensile strength and elongation break of MMMs. Thus, by comprehensive consideration, the best APTES crosslinking time for MMMs is 8 h. The optimal APTES crosslinking condition for MMMs is a 6% concentration for 8 h.

### 3.2. Characterization of PI/HAP MMMs

**Figure 6:**
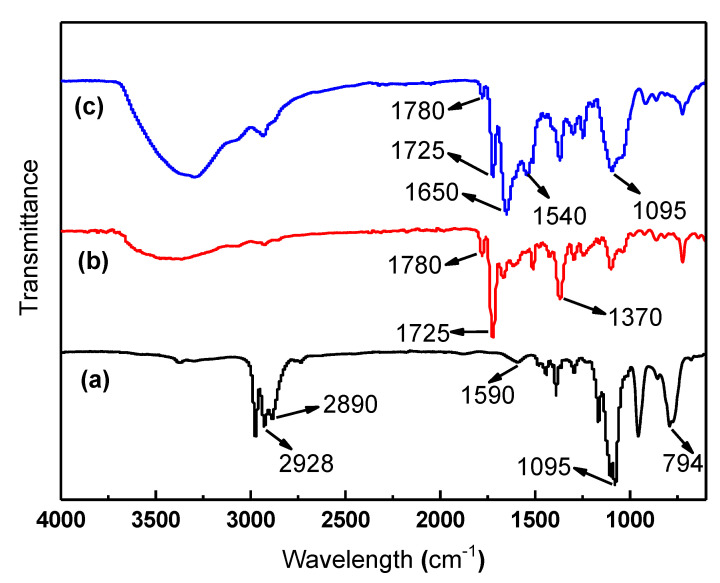
FTIR spectra of (**a**) APTES, (**b**) PI/HAP MMMs and (**c**) crosslinked PI/HAP MMMs.

Crosslinking is an important technology for the improvement of polymer materials’ properties. The corrosion resistance, solvent resistance, plasticization resistance and temperature tolerance can be improved through crosslinking. The PI (P84) membrane was crosslinked with HDA, and the solvent resistance property of the crosslinked PI membrane was enhanced [24]. PI was crosslinked with EDA, and the plasticization resistance property of the crosslinked PI membrane was enhanced [27]. The imide groups of PI molecules react with the amino group of APTES, and PI molecules are crosslinked, as shown in Figure 1. Figure 6 shows the FTIR spectra of APTES, PI/HAP MMMs and crosslinked PI/HAP MMMs. Table 1 shows the FTIR spectra characteristic peaks data of APTES, PI/HAP MMMs and crosslinked PI/HAP MMMs. For APTES, the characteristic peaks at 1095 cm^−1^ and 794 cm^−1^ are the stretching vibration band and bending vibration band of Si–O–Si, respectively, and the characteristic peak at 1590 cm^−1^ is the bending vibration band of –NH_2_. The characteristic peaks at 2928 cm^−1^ and 2890 cm^−1^ are the anti-symmetric and asymmetric stretching bands of –CH_2_– in APTES [30,35]. After PI/HAP MMMs were crosslinked with APTES, the characteristic peaks of the imide group at around 1780 cm^−1^ (symmetric C=O stretching), 1725 cm^−1^ (asymmetric C=O stretching), and 1370 cm^−1^ (C–N stretching) became weakened [36]. Meanwhile, the characteristic peaks of the amide group at 1650 cm^−1^ (C=O stretching) and 1540 cm^−1^ (C–N stretching of C–N– group) appeared [37], and the characteristic peaks of Si–O–Si of APTES at 1095 cm^−1^ appeared [38]. The above change suggests that crosslinked PI/HAP MMMs were successfully prepared. It can be concluded that the imide groups of PI reacted with APTES and that PI molecules were crosslinked with amide bonds of APTES. PI/HAP MMMs were successfully prepared. However, the presence of the characteristic peaks of the imide group suggests that the crosslinking reaction was not completed.

### 3.3. Morphology of PI/HAP MMMs

**Figure 7:**
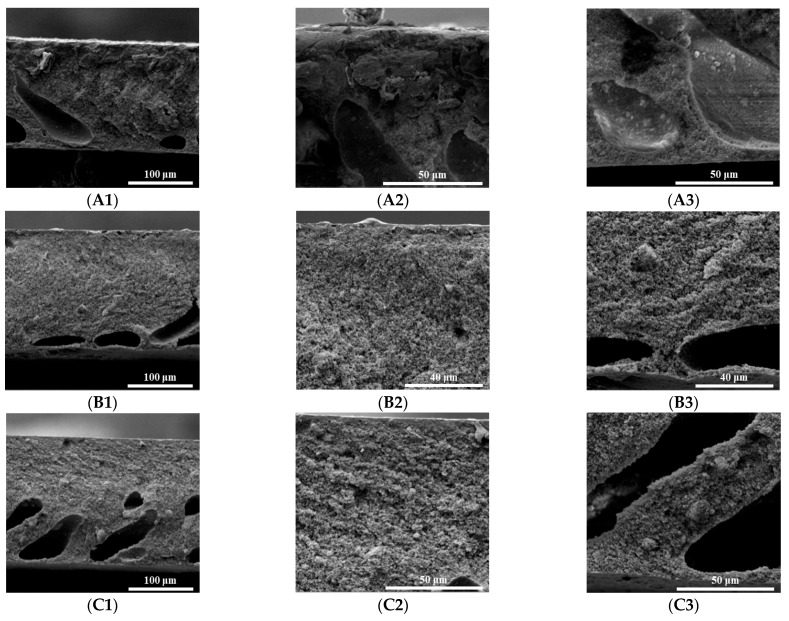
Scanning electron microscope (SEM) micrographs of (**A1**–**A3**) pure PI membrane, (**B1**–**B3**) PI/HAP MMMs and (**C1**–**C3**) crosslinked PI/HAP MMMs. ((**A1**,**B1**,**C1**): cross-section; (**A2**,**B2**,**C2**): top skin layer; (**A3**,**B3**,**C3**): bottom skin layer. 20% HAP was embedded in PI/HAP MMMs).

Figure 7 shows SEM micrographs of the pure PI membrane and PI/HAP MMMs and crosslinked PI/HAP MMMs. The cross-sections of three membranes are finger-like, as shown in Figure 7. The finger-like pores of the pure PI membrane almost run through the whole support layer, and the finger-like pores of PI/HAP MMMs just possess a half support layer. The finger-like asymmetric structure usually formed by using water as a coagulation bath due to a rapid liquid-liquid demixing process during the membrane fabrication, which is common for membrane fabrication by non-solvent induced phase separation (NIPs) [39]. Compared to the high viscosity of PI/HAP dope, the low viscosity of PI dope accelerates the double diffusion process, which results in the formation of large finger-like pores in the support layer of the pure PI membrane. As shown in Figure 7(B1–B3,C1–C3), PI/HAP MMMs and crosslinked PI/HAP MMMs have similar structures, which means that the crosslinking reaction does not change the structure of MMMs. HAP particles could be obviously observed in the support layer of MMMs. The skin layer of MMMs is denser than that of the pure PI membrane because the high viscosity of PI/HAP dope depresses the double diffusion process and dense membrane support forms.

**Figure 8:**
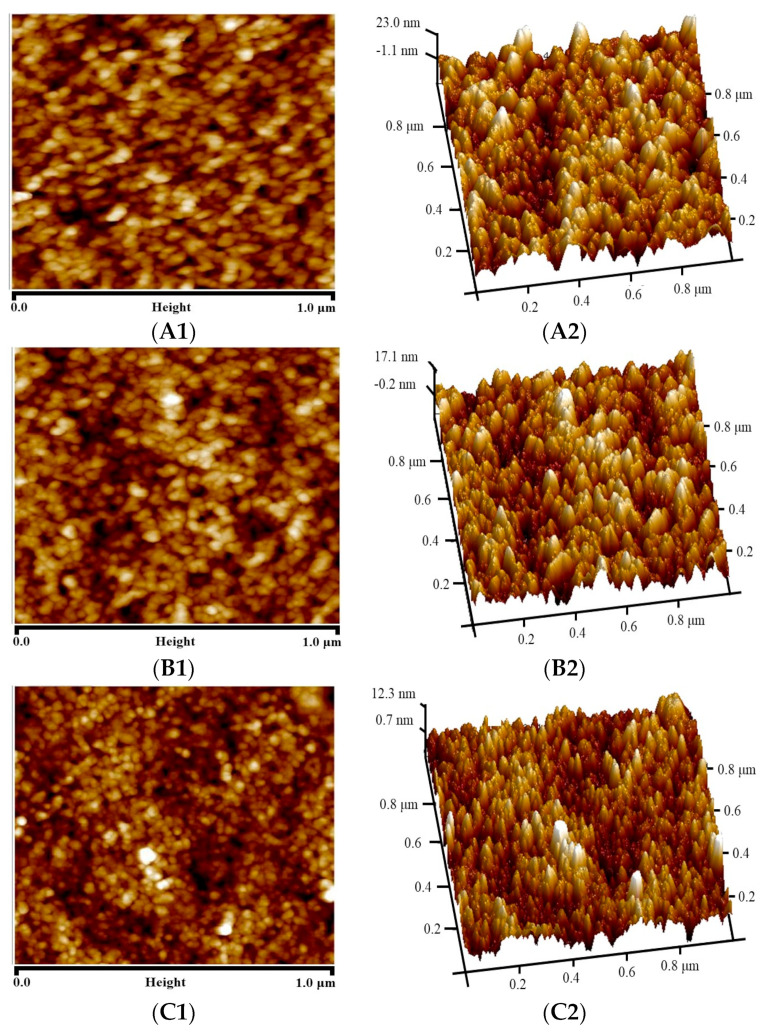
Atomic force microscope (AFM) micrographs of (**A1**,**A2**) pure PI membrane, (**B1**,**B2**) PI/HAP MMMs and (**C1**,**C2**) crosslinked PI/HAP MMMs.

Figure 8 shows AFM micrographs of the pure PI membrane, PI/HAP MMMs and crosslinked PI/HAP MMMs. Table 2 shows the surface roughness parameters of the PI membrane, PI/HAP MMMs and crosslinked PI/HAP MMMs. The bright part is the highest point of the membrane surface, and the dark region is the lowest point of the membrane surface. The average roughness (R_a_), the root mean square roughness (R_q_) and the distance between the highest point and the lowest point (R_max_) are the roughness parameters, which could reflect the surface roughness of membranes. As shown in Figure 8 and Table 2, the surface of the pure PI membrane is coarse, and R_a_, R_q_ and R_max_ are 5.49 nm, 6.86 nm and 51.10 nm, respectively. The surface roughness parameter of MMMs obviously decreases, especially when the MMMs are crosslinked with APTES. The PI solution fixes the position of HAP at the beginning, and the viscosity of the system also limits the Brown movement and gravity sedimentation of HAP particles after HAP particles are homogeneously dispersed in PI dope. That some HAP particles evenly distribute on the surface of MMMs might result in the surface reorganization of PI molecular chains, which leads to the decreases of the surface roughness of MMMs. The MMMs have the lowest surface roughness parameter after they are crosslinked with APTES. PI molecular chains change from a linear structure to a network structure during the crosslinking process, and the distance between PI molecular chains decreases. The surface of crosslinked MMMs is smoother compared to the surface of MMMs, which leads to further decreases of the surface roughness of crosslinked MMMs.

### 3.4. LZ Adsorption of PI/HAP MMMs

#### 3.4.1. LZ Adsorption of MMMs in Organic-Solvent-Aqueous Solutions

**Figure 9:**
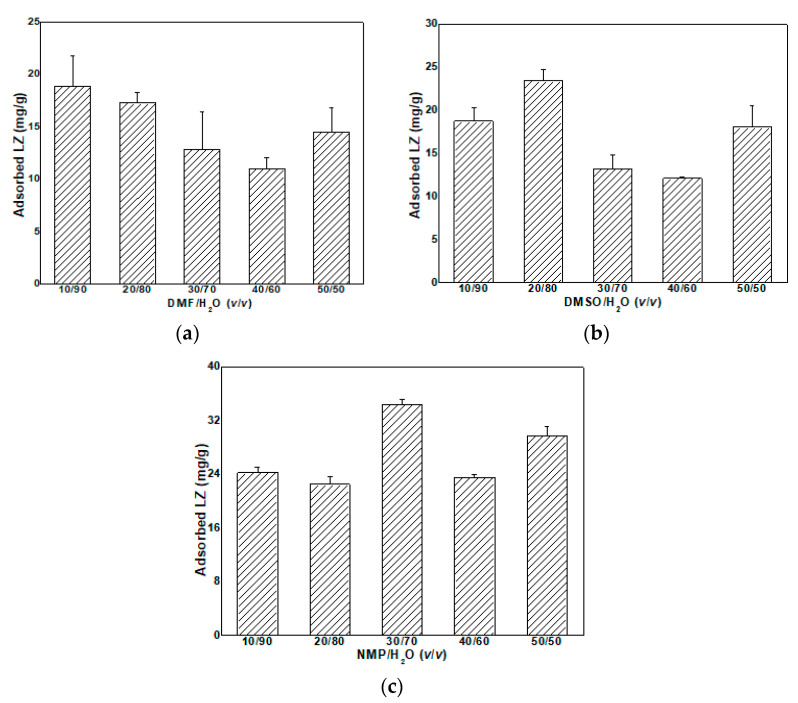
LZ adsorption of MMMs in different organic-solvent-aqueous solutions: (**a**) DMF/H_2_O, (**b**) DMSO/H_2_O, and (**c**) NMP/H_2_O.

Figure 9 shows the LZ adsorption of MMMs in different organic-solvent-aqueous solutions. In the aqueous system, the adsorption is based on electrostatic interactions between the biomolecules and charged particles, whereas in the organic solvents, hydrophobic/hydrophilic interactions play a dominant role [7]. As shown in Figure 9, the adsorption property of MMMs was tested in three different organic-solvent-aqueous solutions, such as DMSO/H_2_O, DMF/H_2_O and NMP/H_2_O. The LZ adsorption capacity respectively reached the maximum (18.9 mg/g, 23.4 mg/g, 34.1 mg/g) when the ratio of DMSO/H_2_O, DMF/H_2_O, and NMP/H_2_O was respectively 10/90 (*v*/*v*), 20/80 (*v*/*v*), and 30/70 (*v*/*v*). The whole LZ adsorption capacity in NMP/H_2_O is the highest among the three organic-solvent-aqueous solutions, which is maybe related to the polarity of the solvent. The solubility and polarity parameter of the three solvents is DMSO (26.7 MPa^1/2^, 16.4 MPa^1/2^) > DMF (24.8 MPa^1/2^, 13.7 MPa^1/2^) > NMP (22.9 MPa^1/2^, 12.3 MPa^1/2^) [40].

LZ is a polarity macromolecule, and the stronger interaction between the LZ macromolecule and solvent happens when the solvent with a higher solubility and polarity parameter is used, which leads to the formation of more entanglements on one LZ molecular chain. LZ is more unfolding in the solvent with a low solubility and polarity parameter, and unfolding LZ is easily adsorbed. In this case, the unfolding degree of LZ in three solvents is DMSO < DMF < NMP. A low solubility and polarity parameter of a solvent is helpful to the LZ adsorption capacity. Therefore, the LZ adsorption capacity of MMMs in three solvents is NMP > DMF > DMSO. There are fewer reports about the adsorption property of LZ in the organic solvents. Kopec et al. reported that the adsorption of LZ onto crosslinked PI/cation exchange particle MMMs was 27.9 mg/g [7], lower than the results obtained in this work (34.1 mg/g). Ro et al. used powderized liposomes to carry LZ, and the loading amount of LZ in liposome was 5.4% at a volume ratio of aqueous to organic of 1:4 [40]. The absolute values cannot be compared to this work because the units of adsorption capacity are different.

#### 3.4.2. LZ Recovery of MMMs

**Figure 10:**
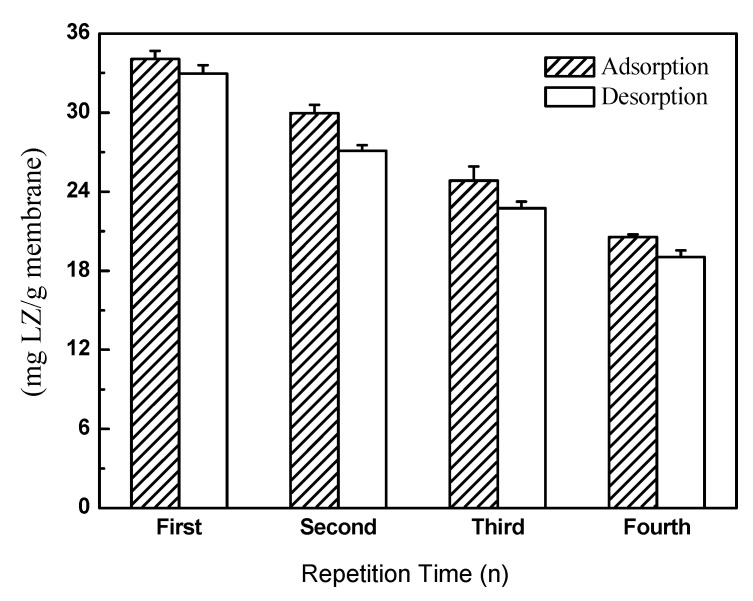
LZ recovery of crosslinked PI/HAP MMMs.

Figure 10 shows the LZ recovery of crosslinked PI/HAP MMMs. As shown in Figure 10, PI/HAP MMMs have a good recovery property. After the LZ adsorption–desorption cycle occurs four times, the LZ adsorption capacity of PI/HAP MMMs decreases from 34.1 mg/g to 20.6 mg/g because of the membrane fouling. The desorption of MMMs decreases from 33.0 mg/g to 19.1 mg/g. The MMMs maintained 60% of their adsorption capacity and 58% of their desorption at the fourth cycle of adsorption and desorption. This means that adsorbed LZ in MMMs is not completely desorbed in every adsorption–desorption cycle and that the number of active sites decreases with increasing cycle times. It is suggested that MMMs are suitable for a repeated application for LZ adsorption.

## 4. Conclusions

PI/HAP MMMs were crosslinked with APTES, and the optimal APTES crosslinking condition for PI/HAP MMMs was a 6% concentration for 8 h. The MMMs were successfully applied for LZ adsorption in an organic-solvent-aqueous solution. The LZ adsorption capacity reached the maximum (34.1 mg/g) in an NMP aqueous solution. The MMMs had a high desorption capacity and recovery ability after the fourth adsorption–desorption cycle. PI/HAP MMMs have a potential application in the field of protein and enzyme purification in organic solvent systems.

## Figures and Tables

**Table 1:** FTIR spectra characteristic peaks data of 3-aminopropyl triethoxysilane (APTES), polyimide/hydroxyapatite (PI/HAP) MMMs and crosslinked PI/HAP MMMs.

APTES		PI/HAP MMMs		Crosslinked PI/HAP MMMs	
Wavelength (cm^−1^)	Group	Wavelength (cm^−1^)	Group	Wavelength (cm^−1^)	Group
794	bending vibration band of Si–O–Si	1370	C–N stretching	1095	stretching vibration band of Si–O–Si
1095	stretching vibration band of Si–O–Si	1725	asymmetric C=O stretching	1540	C–N stretching of C–N– group
1590	bending vibration band of –NH_2_	1780	symmetric C=O stretching	1650	C=O stretching
2890	asymmetric stretching bands of –CH_2_–			1725	asymmetric C=O stretching
2928	anti-symmetric asymmetric stretching bands of –CH_2_–			1780	symmetric C=O stretching
References [30,35]		Reference [36]		References [37,38]	

**Table 2:** Surface roughness parameters of PI membrane, PI/HAP MMMs and crosslinked PI/HAP MMMs.

Roughness Parameter	PI Membrane	MMMs	Crosslinked MMMs
R_a_ (nm)	5.49	4.07	2.54
R_q_ (nm)	6.86	5.07	3.25
R_max_ (nm)	51.10	43.20	32.80

## Data Availability

Data available in a publicly accessible repository.
